# Searching for Frataxin Function: Exploring the Analogy with Nqo15, the Frataxin-like Protein of Respiratory Complex I from *Thermus thermophilus*

**DOI:** 10.3390/ijms25031912

**Published:** 2024-02-05

**Authors:** Davide Doni, Eva Cavallari, Martin Ezequiel Noguera, Hernan Gustavo Gentili, Federica Cavion, Gustavo Parisi, Maria Silvina Fornasari, Geppo Sartori, Javier Santos, Massimo Bellanda, Donatella Carbonera, Paola Costantini, Marco Bortolus

**Affiliations:** 1 Department of Biology, University of Padova, 35121 Padova, Italy; davide.doni@unipd.it (D.D.); federica.cavion@unipd.it (F.C.); 2 Grenoble Alpes University, CNRS, CEA, INRAE, IRIG-LPCV, 38000 Grenoble, France; 3 Department of Physiology and Molecular and Cellular Biology, Institute of Biosciences, Biotechnology and Translational Biology (iB3), Faculty of Exact and Natural Sciences, University of Buenos Aires, Intendente Güiraldes 2160, Buenos Aires C1428EG, Argentina; mnoguera.unq@gmail.com (M.E.N.); hernangentili77@gmail.com (H.G.G.); javiersantosw@gmail.com (J.S.); 4 Institute of Biological Chemistry and Physical Chemistry, Dr Alejandro Paladini (UBA-CONICET), University of Buenos Aires, Junín 956, Buenos Aires 1113AAD, Argentina; 5 Department of Science and Technology, National University of Quilmes, Roque Saenz Peña 352, Bernal B1876BXD, Argentina; gusparisi@gmail.com (G.P.); silvina333@gmail.com (M.S.F.); 6 Department of Biomedical Sciences, University of Padova, 35121 Padova, Italy; geppo.sartori@unipd.it; 7 Department of Chemical Sciences, University of Padova, 35131 Padova, Italy; massimo.bellanda@unipd.it (M.B.); donatella.carbonera@unipd.it (D.C.); 8 Consiglio Nazionale delle Ricerche Institute of Biomolecular Chemistry, 35131 Padova, Italy

**Keywords:** Friedreich’s ataxia, Frataxin, Nqo15

## Abstract

Nqo15 is a subunit of respiratory complex I of the bacterium *Thermus thermophilus*, with strong structural similarity to human frataxin (FXN), a protein involved in the mitochondrial disease Friedreich’s ataxia (FRDA). Recently, we showed that the expression of recombinant Nqo15 can ameliorate the respiratory phenotype of FRDA patients’ cells, and this prompted us to further characterize both the Nqo15 solution’s behavior and its potential functional overlap with FXN, using a combination of in silico and in vitro techniques. We studied the analogy of Nqo15 and FXN by performing extensive database searches based on sequence and structure. Nqo15’s folding and flexibility were investigated by combining nuclear magnetic resonance (NMR), circular dichroism, and coarse-grained molecular dynamics simulations. Nqo15’s iron-binding properties were studied using NMR, fluorescence, and specific assays and its desulfurase activation by biochemical assays. We found that the recombinant Nqo15 isolated from complex I is monomeric, stable, folded in solution, and highly dynamic. Nqo15 does not share the iron-binding properties of FXN or its desulfurase activation function.

## 1. Introduction

Frataxin (FXN), a ubiquitous and highly conserved mitochondrial protein, is associated with Friedreich’s ataxia (FRDA), a cardio- and neurodegenerative disease characterized by progressive gait and limb ataxia, dysarthria, loss of proprioception and coordination, diabetes mellitus and hypertrophic cardiomyopathy, which represents the primary cause of death [1,2,3,4]. FRDA is a genetic disorder, due to the abnormal expansion of the GAA trinucleotide repeat in the first intron of the *FXN* gene, leading to a severe protein deficiency in cells [1,5]; moreover, a small percentage of patients (~4%) are compound heterozygous for the expansion of one *FXN* allele and for a point mutation on the other, which has deleterious effects on both protein stability and functionality [6,7]. The pathophysiological mechanisms underlying the FRDA disease are still not fully clarified; however, some specific biochemical hallmarks are common, such as the general dysregulation of cellular iron homeostasis [8], higher susceptibility to oxidative stress [9] and an impairment in the biogenesis of heme centers and Fe-S clusters [10], key redox cofactors involved in different and crucial metabolic pathways.

Understanding the still elusive role of FXN would offer a substantial contribution to the development of a cure for FRDA: different roles have been proposed for the protein and all are directly or indirectly related to iron trafficking or metabolism. To date, it is widely accepted that FXN is involved in the biosynthesis of Fe-S clusters [11,12,13,14,15,16], a pathway that occurs in mitochondria and is performed by a multiprotein complex composed of cysteine desulfurase (NFS1), the iron-sulfur cluster biogenesis desulfurase interacting protein (ISD11), the acyl carrier protein (ACP) and the iron-sulfur cluster assembly enzyme (ISCU), the scaffold protein upon which the clusters are synthesized [17,18]. Although it has been proven that FXN acts as an allosteric activator for the Fe-S cluster assembly machinery [19,20,21,22,23,24], it is not yet known how the iron-binding capacity of the protein is related to this process. In this regard, it has been determined that FXN binds both Fe^2+^ and Fe^3+^ at physiologically relevant concentrations through conserved Asp and Glu residues exposed on the protein surface; due to this property, it has also been proposed that FXN could act as a protein for iron delivery or storage in the cell [25,26,27,28,29,30,31,32,33,34,35,36,37].

The involvement of the protein in redox homeostasis is still unclear as well, but it has been demonstrated that human FXN interacts, at least in vitro, with mitochondrial superoxide dismutase (SOD2), a key enzyme involved in the defense against oxidative stress [38].

We have recently shown that, in healthy human cells, FXN is associated with mitochondrial cristae, the subcompartment that houses the respiratory chain [39]. The raison d’être of this enrichment lies in the ability of FXN to functionally interact with respiratory complexes I, II and III, as demonstrated by combining different experimental approaches [40]. Remarkably, the decrease in FXN in FRDA cells has been shown to lead to the more severe impairment of complex I than complexes II and III, suggesting that FXN could have a specific role in the stabilization and/or functioning of the first complex of the respiratory chain. The hypothesis of a functional interaction between FXN and mitochondrial complex I has been strengthened by an interesting finding relative to respiratory complex I from the bacterium *Thermus thermophilus*. The crystal structure of the hydrophilic domain of *T. thermophilus* complex I reveals a subunit, called Nqo15, which is additional to the canonical 14 catalytic core subunits highly conserved from bacteria to mammals, and which is structurally analogous to human FXN and its protein family [41,42]. Nqo15 adopts the FXN fold, and a comparison of FXN homologues from different organisms reveals a high degree of structural similarity, all sharing an α-β sandwich motif with two α-helices packing six- to seven-stranded β-sheets [25,26,29,30]. Interestingly, a FXN homologue based on sequence similarity has not yet been identified in *T. thermophilus*, pointing to Nqo15 as a possible evolutionarily distant member of the FXN protein family. In this regard, it should be mentioned that the *Nqo15* gene locus is separated from the *nqo* operon, where the genes encoding the 14 conserved core subunits of complex I are grouped. However, although it shares the peculiar folding of FXN, Nqo15 shows a low degree of sequence identity, raising the question of whether the protein displays functional properties shared by all members of the FXN family, such as iron-binding and the enhancement of Fe-S cluster formation. Indeed, we have shown that the expression of a recombinant Nqo15 is capable of ameliorating the respiratory phenotype of FRDA patients’ cells [40], and this prompted us to further characterize Nqo15.

In the present work, by performing an in silico analysis of Nqo15 and combining different spectroscopic approaches on the recombinant protein, we explore the similarity and degree of functional overlap between Nqo15 and human FXN to provide new information on the bacterial protein and, by extension, on the relationship between human FXN and respiratory complex I.

## 2. Results

### 2.1. Searching for Nqo15 and FXN Homology

**Figure 1:**
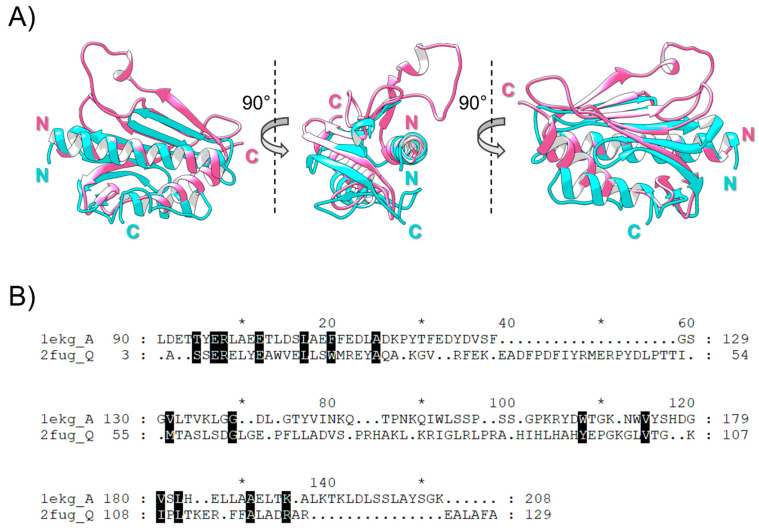
Comparison between human FXN and *T. thermophilus* Nqo15. (**A**) Different views of the structural alignment between human FXN (PDB.ID: 1EKG_A; cyan) and Nqo15 (PDB.ID: 2FUG_Q; pink). Estimated alpha-C RMSD = 2.34 Å and sequence identity percentage is ~7%. N-terminal and C-terminal regions have been highlighted in the appropriate color. Starting from the left, each one of the following views has been in turn rotated 90° to the right. (**B**) Sequence alignment derived from structural superposition colored in conservation mode; asterisks denote the odd tens in the sequence.

To establish homology relationships between FXN and Nqo15, evidence of sequence and/or structural similarity should be obtained. Sequence similarity searches with BLAST, PSIBLAST and HMMER using FXN or Nqo15 as queries did not retrieve significant cross-similar proteins belonging to both protein families. FXN searches retrieved a large number of proteins (~10,000 proteins), showing a wide distribution across metazoa, fungi, yeasts, plants and eubacteria. On the other hand, searches with Nqo15 retrieved proteins exclusively belonging to the Deinococcota phylum (~190 proteins), a group of aerobic extremophile bacteria. However, the SCOP database, devoted to the structural-based classification of proteins, includes the superfamily “Frataxin/Nqo15-like” (SCOP ID: 3001840) with two families: (1) the “Frataxin-like” family (SCOP ID: 4001566), comprising six members (i.e., FXN coming from *Saccharomyces cerevisiae*, *Homo sapiens*, *Psychromonas ingrahamii*, *Chaetomium thermophilum*, *Escherichia coli* and *Burkholderia cenocepacia*, see Table 1); (2) the “Nqo15-like” family (SCOP ID: 4003855), containing structures coming from *T. thermophilus*, mainly represented by Nqo15 structures (Table 1). Additionally, structures of Nqo15 were retrieved with statistical significance (Z-score > 7.0), starting with a structural similarity search against the PDB databases using FXN as a query (PDB.ID: 1EKG chain A structure). The structural alignment of FXN and Nqo15 gives an alpha-C RMSD = 2.34 Å with an overall sequence identity percentage of ~7% (Figure 1). Additional proteins with FXN-like folds were also retrieved from Dali searches; in particular, the YdhG protein from *Bacillus subtilis* was also found with an RMSD = 3.1 Å and Z-score = 4.9 (PDB.ID: 2OC6 chain A) [43,44]. YdhG belongs to the SCOP superfamily YdhG-like (SCOP ID: 4002604) with two additional members, the DUF1801 domain-containing protein from *Lactobacillus paracasei* (PDB.ID: 2I8D chain A) and the BH2032 protein (PDB.ID: 2KL4 chain A) from *Bacillus halodurans.*

**Figure 2:**
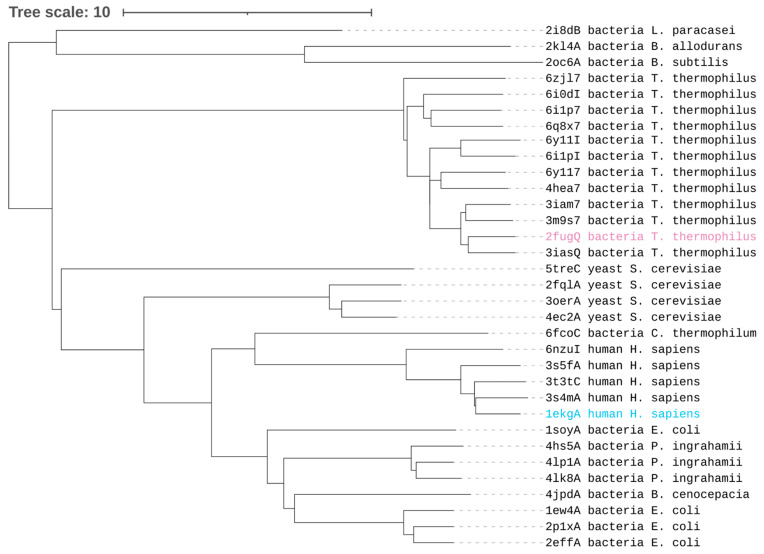
Dendrogram of structure similarity obtained using an All vs. All alignment for representatives of FXN-like folds. Distances between structures are derived from their respective Z-scores of structure similarity. Entries’ names contain PDB.IDs with the corresponding chain, followed by a short taxonomic name and species name. The structures of Nqo15 (pink) and FXN (cyan) used in Figure 1 are highlighted.

To further explore the structural similarities between Nqo15 and representatives of FXN-like folds, we used the Dali server to obtain a similarity dendrogram based on All vs. All structural comparisons. Using the representative structures deposited in the “Frataxin/Nqo15-like” (SCOP ID: 3001840) superfamily (33 structures and conformers) and “YdhG-like” superfamily (SCOP ID: 4002604) (3 structures) (see Table 1), we obtained the dendrogram shown in Figure 2. In this dendrogram, structural differences are measured as distances between Dali Z-scores (where higher values indicate higher structural similarities). Different structures for the same protein obtained under different conditions are useful in estimating the conformational diversity of the protein [44]. Figure 2 shows that Nqo15 forms a separate cluster from the rest of the frataxin families, from those coming from yeast, human and bacteria. According to this clustering, the closest group to human FXN contains FXN-like coming from the bacteria *E. coli*, *B. cenocepacia* and *P. ingrahamii*. It is also shown that the YdhG-like family forms an outgroup, indicating larger structural differences.

### 2.2. Structural Integrity, Solubility and Stability of Standalone Nqo15

**Figure 3:**
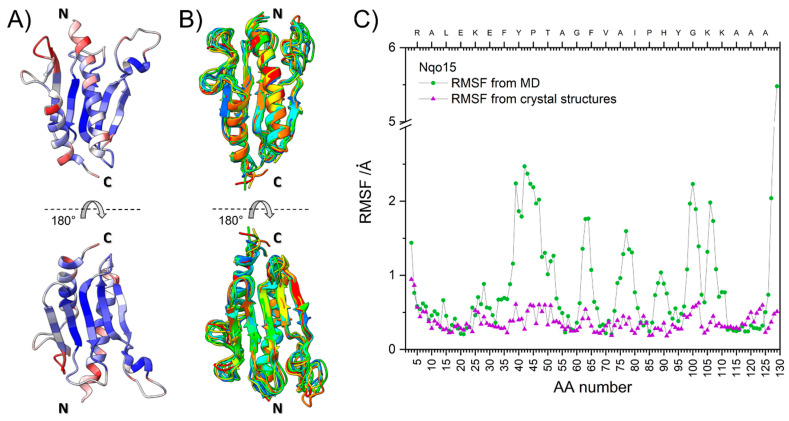
Molecular dynamics of Nqo15. (**A**) Two views of the Nqo15 structure extracted from PDB.ID: 2FUG_H colored by B-factor (from low to high, blue–white–red); the N- and C-termini are highlighted. (**B**) The same views of the coarse-grained MD models of Nqo15 obtained using CABS-flex 2.0, where each model has a different color; the N- and C-termini are highlighted. In the bottom view, the N-terminus is obscured by the loop. (**C**) The root-mean-square fluctuations (RMSF, in Å) for Nqo15 obtained from the MD simulations (green) and the average obtained from the comparison of all available crystal structures of Nqo15 with the one from PDB.ID: 2FUG_H (purple); the residue number is on the bottom axis, the residue one-letter code on the top axis.

We first analyzed the crystal structure of Nqo15 isolated from the rest of complex I (chain H of PDB.ID: 2FUG) by performing an MD analysis [45]. The results are reported in Figure 3, where two views of the crystal structure colored by the B factor [46] are shown (Figure 3A), the same views of the ensemble structures obtained from the MD colored by structure (Figure 3B) and the plot of the root mean square fluctuation (RMSF) of each residue calculated in the MD ensemble (Figure 3C, green). Parallelly, we also obtained the average RMSF obtained by comparing all Nqo15 structures from *T. thermophilus* reported in the dendrogram (Figure 3C, purple). MD simulations showed that the N-terminal portion (4–35) is remarkably stable, both the long α-helix and the first β-strand. The region with the highest dynamics (35–55) is the long loop that is absent in FXN; this portion of the protein in the crystal structure has medium–low B-factors, suggesting that it is somewhat stabilized by the contacts with the other subunits of complex I, obviously absent in solution. The other dynamic peaks belong to the β-strand terminals and the loop connecting each strand. Finally, the C-terminal helix shows high fraying in the last four residues. The comparison of the RMSF data shows that, in general, the portions of the protein with greater variability in the crystal structures correspond to those with a greater fluctuation in the MD ensemble. Two notable exceptions are found in the C-terminus, significantly more dynamic for the protein alone than in the crystal structure, and the 35–55 loop, which shows the most pronounced increase in RMSF from the crystal to the isolated protein. As expected, inherently flexible regions, such as unstructured loops, show increased flexibility in solution compared to the crystal structure, but the overall fold of the protein is not grossly distorted. The structure of isolated Nqo15 is more dynamic than that of human FXN, as determined by the comparison of the MD simulations [38]: aside from the C-terminus, the RMSF of FXN peaks at 2 Å and is, on average, lower than 1.5 Å, whereas several regions of Nqo15 have an RMSF of 2 Å or higher.

**Figure 4:**
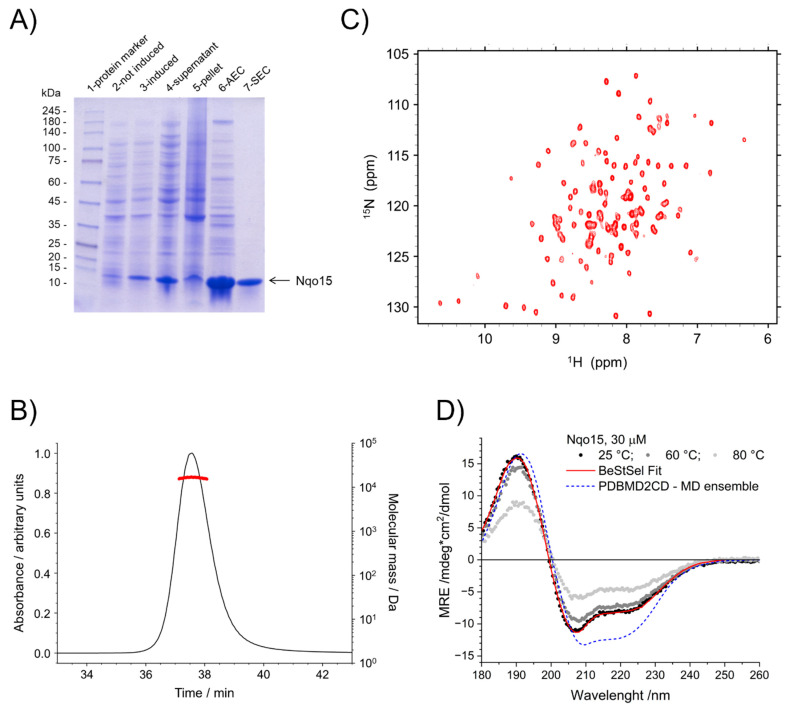
Expression, purification and structural characterization of Nqo15. (**A**) Expression and purification of recombinant *T. thermophilus* Nqo15 as examined by SDS-PAGE and Coomassie blue staining. Lane (2) and lane (3) refer to the bacterial culture before and after the induction with IPTG, respectively. Lane (4) and lane (5) correspond to the soluble and insoluble fraction of the total cellular lysate. In lane (6), the protein pool after anionic exchange chromatography (AEC) is loaded. Lane (7) represents the pool of purified fractions containing Nqo15 obtained by size-exclusion chromatography (SEC). Lane (1): molecular weight protein ladder. (**B**) SEC-MALLS analysis of the aggregation state of Nqo15, protein concentration of 260 µM, elution profile from the Superose 12 column monitored at 280 nm (black line) and associated molecular mass (red dots). (**C**) ^1^H-^15^N SOFAST-HMQC spectrum of Nqo15, protein concentration 50 µM. (**D**) CD spectrum of Nqo15 (black dots—25 °C; dark grey dots—60 °C; light grey dots—80 °C), protein concentration 30 µM, CD spectrum analysis (red line) using the BeStSel webserver on the CD data, CD spectrum prediction (blue dashed line) using the PDBMD2CD webserver based on the MD structural ensemble calculated starting from PDB.ID: 2FUG_H.

As mentioned above, Nqo15 is an integral part of the hydrophilic domain of *T. thermophilus* complex I, so its existence as an individual protein in the organism has not been reported. To address whether Nqo15 could be structurally stable when isolated from the complex and whether it could retain some of the functional properties displayed by human FXN, we overexpressed it in *E. coli* and purified the protein, as described in detail in Materials and Methods. The heterologous expression and purification were checked by SDS-PAGE and Coomassie blue staining, showing, in each lane, a band of expected molecular weight (Figure 4A). It is worth noting that Nqo15 was almost completely found in the soluble fraction after cell lysis (Figure 4A, compare lanes 4 and 5), suggesting a weak tendency of the protein to aggregate, at least in vitro. The aggregation state of Nqo15 in solution was also demonstrated by static light scattering experiments, which produced a single peak corresponding to a molecular mass of 16.2 ± 0.3 kDa (Figure 4B). The small discrepancy from what was expected (14.6 kDa) is probably due to differences in the native extinction coefficient from that estimated from the sequence, the latter used for data processing. Furthermore, the aggregation state does not change depending on the salt concentration: the same mass was detected for experiments run at a 50 mM or 200 mM NaCl concentration.

Although obtained in pure and soluble form, we investigated whether Nqo15 also retained its structure, compared to that reported in *T. thermophilus* complex I. Since NMR spectroscopy is a powerful tool for the investigation of the structure and dynamics of small proteins, we performed a SOFAST-HMQC experiment [47] of ^15^N-labeled Nqo15. The two-dimensional ^1^H-^15^N correlation spectrum of Nqo15, reported in Figure 4C, reveals a good dispersion of signals, indicating a well-folded, non-aggregated protein. However, the presence of some weak peaks indicates that some regions of the protein undergo conformational broadening as a result of increased backbone dynamics. The ^1^H-^15^N SOFAST-HMQC does not allow the full assignment of all amino acids. However, NMR confirms that Nqo15 is stable, does not aggregate and retains a high degree of globular structure when isolated from its partner subunits.

Circular dichroism (CD) is a valuable tool for the rapid determination of protein folding properties and secondary structure content in terms of α-helices, β-strands and turns. To infer proteins’ secondary structures, the range of wavelengths where the peptide bonds absorb (ideally between 180 and 260 nm) is usually explored. The CD spectrum of Nqo15 at 25 °C is reported in Figure 4D in black dots. The spectrum at 25 °C shows a typical profile of well-structured proteins characterized by the presence of both α-helix and β-sheet motifs. We used the BeStSel webserver [48] to analyze the CD spectrum and estimate the content of the secondary structure: α-helix = 19%, β-strand = 25%, turn = 15%. An analysis of the secondary structure content based on the crystal structure using the BeStSel tools yields the following: α-helix = 33%, β-strand = 29%, turn = 8%. The discrepancy in the secondary structure content of the experimental data suggests that, in solution, there may be some loss of secondary structure, as already evidenced by the comparison between the MD ensemble and the crystal structures. Therefore, to obtain an idea of whether the protein retains the same folding in solution as shown in the structure of complex I in *T. thermophilus*, the CD spectrum was also predicted on the basis of the MD ensemble models using the PDBMD2CD webserver [49] (blue dashed line in Figure 4D): the program calculates one spectrum for each MD-generated structure and then outputs the averaged spectrum reported here. The calculated ensemble spectrum matches the experimental data reasonably well; the analysis reports the following secondary structure composition: α-helix = 27 ± 3%, β-strand = 16 ± 3%, turn = 11 ± 3%. The CD spectrum was also acquired at higher temperatures to explore its stability and potential unfolding: sample high-temperature data, at 60 °C and 80 °C, are shown in Figure 4D in grey dots. The CD does not show any variation up to 40 °C; at 60 °C and 80 °C, the spectral shape is mostly retained, suggesting that only minimal unfolding takes place, but the spectrum loses 23% to 60% of its maximum intensity, suggesting protein precipitation. As a further indication of protein loss, lowering the temperature back to 25 °C does not restore the initial intensity.

Nqo15’s stability in solution was also monitored by measuring the intrinsic tryptophan fluorescence of the protein as a function of time at 15 °C. The fluorescence of Nqo15 ([Nqo15] = 2.2 μM) gradually decreased to a loss of 8% fluorescence after two hours, and afterward remained stable for up to four and a half hours. The purified protein is also amenable to freezing and thawing without any significant precipitation or loss of secondary structure, as monitored by UV–Vis absorption and CD performed on multiple protein aliquots.

Together, the NMR, CD and MD results indicate that the protein is much more dynamic in solution than in the protein complex but is monomeric, retains its globular folding and is stable in solution.

### 2.3. Nqo15 Does Not Bind Iron

**Figure 5:**
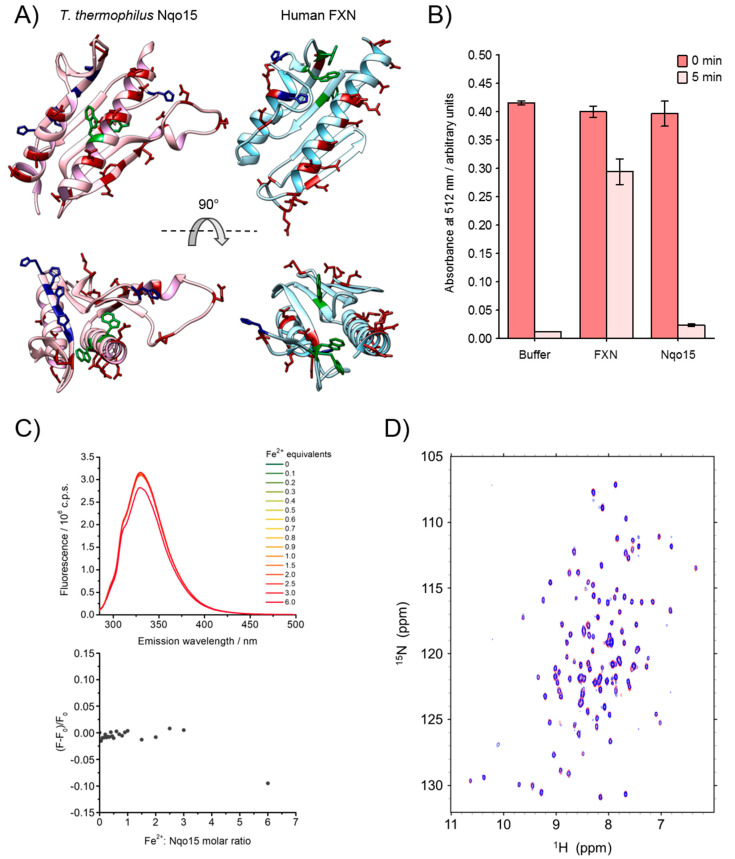
Iron-binding of Nqo15. (**A**) Different views of Nqo15 (PDB.ID = 2FUG_H; pink) and human FXN (PDB.ID = 1EKG_A; cyan) with key amino acids color-coded: Asp/Glu—red; His—blue; Trp—green. (**B**) Iron (III)-binding capability assay. Iron (III) was added in a 5-molar excess to protein samples (50 μM) or buffer alone at neutral pH and the iron content was quantified immediately (0 min) or after 5 min of centrifugation at 25 °C (5 min). Soluble iron was quantified using 1,10-phenantroline and measuring absorbance at 512 nm. (**C**) Trp fluorescence spectra of Nqo15 at increasing amounts of Fe^2+^ (on the top) and fluorescence quenching by Fe^2+^ calculated at λ_max_ (on the bottom). Protein concentration 2.2 µM, 15 °C, λ_exc_ = 280 nm, λ_max_ = 330 nm. (**D**) Superposition of ^1^H-^15^N SOFAST-HMQC spectra of 50 μM Nqo15 in absence (red) and in presence of 4 equivalents of Fe^2+^ (blue).

FXN is known to bind iron in vitro (in both the +2 and +3 oxidation states). Here, we explored the iron-binding propensity of Nqo15 to verify whether it shares this aspect of FXN. FXN binds iron using triplets of negatively charged residues (Asp or Glu); we expect that if Nqo15 shares the same iron-binding as FXN, it would interact with iron through negatively charged residues as well. Nqo15 has as many exposed, negatively charged residues as FXN (Nqo15, 12 Glu and 6 Asp; FXN, 9 Glu and 10 Asp), but none of them are arranged in close triplets, as in those of FXN, although several are arranged as couples that are close enough to each other to possibly serve as iron ligands (see Figure 5A, Asp/Glu—red).

We used a previously described assay to assess whether Nqo15 is capable of maintaining iron in a bioavailable form [50]. In the assay, the protein was incubated at a neutral pH in the non-chelating buffer HEPES with iron (III): at this pH, iron (III) is highly insoluble unless it is bound to the protein. After incubation, the samples were centrifuged, and the soluble iron was quantified by the 1,10-phenanthroline method. As shown in Figure 5B, after 5 min (the shortest time tested), all iron was precipitated for both the buffer alone and Nqo15 samples, while FXN retained about four equivalents of iron.

Since no Fe^3+^ binding was observed in the above-mentioned assay, we also explored the ability of Nqo15 to bind Fe^2+^ iron by monitoring the quenching of the intrinsic Trp fluorescence of Nqo15 in increasing amounts of Fe^2+^ ions, in analogy to what was previously done with FXN [37]. FXN possesses three Trp residues, while Nqo15 has only two (see Figure 5A, highlighted in green): residue Trp13 is buried between the N-terminal α-helix and the β-sheet, while the Trp19 side chain is exposed to the solution. However, no fluorescence quenching was observed in substoichiometric or stoichiometric amounts of Fe^2+^ up to three equivalents (Figure 5C); only in the presence of a large molar excess of iron (up to six equivalents), some quenching could be observed, but, under these conditions, it is likely to be ascribed to a non-specific collisional mechanism favored by the negative charges of acid residues close to Trp19 that attract positively charged iron cations. However, we must note that Nqo15 is highly basic (pI of 9.1 and net charge of approximately +1.5 at pH 7.0) compared to FXN, which is acidic (pI of 4.6 and net charge of approximately −5 at pH 7.0).

Since NMR has previously been used to detect iron-binding by FXN [25,29,36], we also performed NMR titration to verify the effects of Fe^2+^ to Nqo15. Although the addition of Fe^2+^ to FXN caused a clear shift and/or severe broadening of several NMR peaks [36], under similar conditions (i.e., 50 μM protein and 200 μM iron), no notable effects were observed in the SOFAST-HMQC of Nqo15 (Figure 5D).

### 2.4. Nqo15 Does Not Activate Human Cysteine Desulfurase

**Figure 6:**
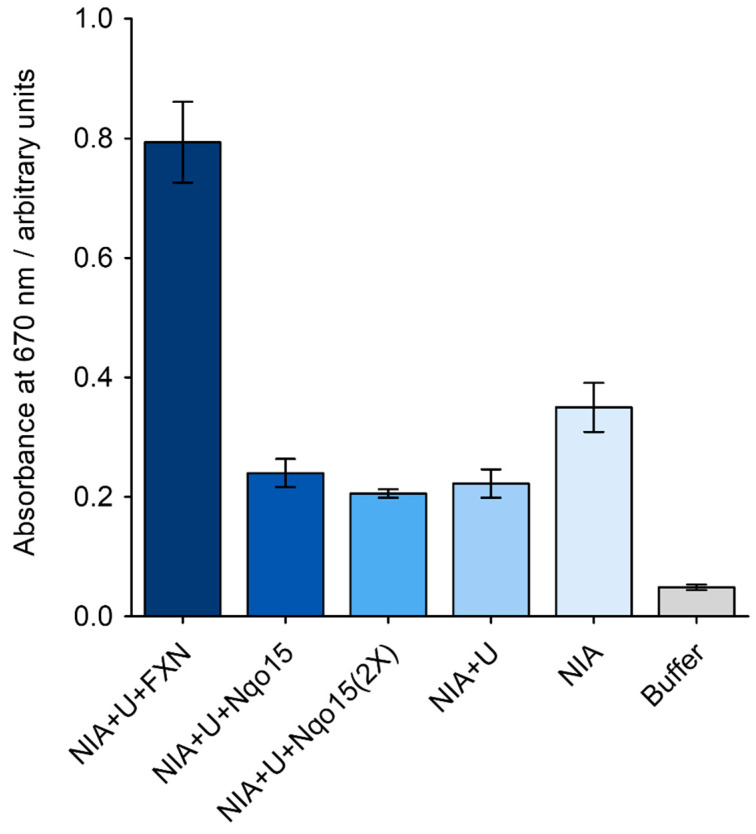
Desulfurase activation assay. Enzymatic activity was followed by the methylene blue method, using 1 μM of NIA (i.e., NFS1/ACP-ISD11 complex) and 3 μM of ISCU (indicated as U). PLP (10 μM), DTT (2 mM) and ferrous iron (1 μM) were present in the reaction media. Nqo15 and FXN were used at 3 μM each (except for the 2X experiment, where Nqo15 was 6 μM). The reaction was started by the addition of 1 mM L-Cys. After incubation (30 min at room temperature), the level of SH_2_ release was revealed with DMPD and the absorbance at 670 nm was measured. Values represent the mean ± SD.

We next focused on the well-documented role of FXN in enhancing the activity of the mitochondrial cysteine desulfurase complex [51]. This complex consists of the cysteine desulfurase NFS1, the stabilizing partners ACP-ISD11 and the scaffolding protein ISCU. We investigated whether Nqo15 is also capable of activating desulfurase by incubating the purified complex of NFS1 and ACP-ISD11 (designated as NIA) and the purified ISCU with FXN or Nqo15. After the addition of L-Cys, the hydrogen sulfide generated was quantified using the methylene blue method [52,53]. As shown in Figure 6, an approximately twofold increase in activity was observed after FXN addition, while no enhancement was observed when Nqo15 was introduced at an equivalent or double molar concentration compared to FXN. Instead, we observed a slight decrease in activity when Nqo15 was added compared to NIA, similar to the decrease observed when NIA was incubated with ISCU (an effect previously described [54]).

## 3. Discussion

The depletion of FXN is critical for the onset and progression of FRDA disease and, although the body of literature on FXN supports the hypothesis of FXN as a pleiotropic protein involved in multiple processes, its precise role in the mitochondrial pathophysiology has not been definitively clarified. Starting from the strikingly similar fold of FXN and Nqo15, a subunit of respiratory complex I of *T. thermophilus*, we explore whether the two proteins share common functions to gain further insight into human FXN. As it has been established, the structures of homologous proteins are more conserved than their sequences during evolution [55], and this observation makes remote homologue detection possible [56]. Human FXN and *T. thermophilus* Nqo15 share high structural similarity, as we confirm in the present work by Dali structural alignment (Figure 1 and Figure 2), and this evidence strongly suggests a meeting point in terms of putative biological functions. The interesting speculation of the separation/addition of Nqo15 from/to respiratory complex I during evolution is corroborated by two observations: (1) Nqo15 is stable and monomeric in solution and maintains its fold even when isolated from complex I (Figure 4); (2) the dynamic residues in solution are also those with the greatest variability in the entire protein complex (Figure 3).

In addition to their structural similarity, the possibility of analogous functions between human FXN and Nqo15 is reinforced by other recent findings reported by our group. We have shown that the impairment of mitochondrial function observed in FRDA patients’ cells could be correlated with the displacement of FXN from the cristae to the matrix, leading to a loss of interaction between FXN and the respiratory chain [39]. The deficiency of FXN in human cells has been shown to mainly impact mitochondrial complex I [40], which, among the respiratory complexes, has a higher turnover rate and is particularly prone to intrinsic degradation and oxidative damage [57]. In this regard, it has been observed that the exogenous expression of recombinant Nqo15 in FRDA patients’ cells is able to ameliorate the respiratory phenotype [40]; the extent of this effect is comparable to what is observed by re-expressing FXN in these cells, indirectly supporting the hypothesis that FXN could play a key role in mitochondrial complex I proteostasis.

The role of the two proteins in respiratory complex I finds some biological context in the two proposed functions of Nqo15 by Sazanov L. and Hinchliffe P. [41]. The first hypothesized function is that, interacting with adjacent subunits (i.e., Nqo1, Nqo2, Nqo3, Nqo4 and Nqo9), Nqo15 could stabilize the terminal part of the hydrophilic domain. As stated by the authors, the lack of Nqo15 would make this region quite narrow compared to the rest of the structure, compromising the structural stability of the peripheral arm of the complex. The second hypothesis is that Nqo15 could act as an iron-binding protein, storing the metal and ensuring the regeneration of the adjacent Fe-S clusters of the complex. In this regard, a closer inspection of the crystallographic structure reveals that Nqo15, by the interaction through its β-sheet with the side of the complex, leads to the formation of a hydrophilic channel constituted by six histidine residues (see Figure 5A, in blue), four of them provided by Nqo15 and located on a single β-strand (i.e., _15_His^90^, _15_His^92^, _15_His^94^ and _15_His^96^) and the other two provided by subunits Nqo1 (_1_His^350^) and Nqo3 (_3_His^208^). Interestingly, it has been proposed that these residues could be involved in the coordination and delivery of cations towards the end of the channel, where a negatively charged protein surface, close to the region that accommodates Fe-S clusters N1a and N3, could act as a metal-binding site. In fact, divalent cations were found to bind in this acidic groove, including the Mn^2+^ ion, which, due to its physicochemical properties, can resemble Fe^3+^ [58]. The hypothesis of Nqo15 as an iron-binding protein is also strengthened by the structural similarity between Nqo15 and the members of the FXN family, which are well known to bind iron, also supported by the lack of an FXN homologue in *T. thermophilus*, which could exert an analogous function. In the present work, we investigate this assumption, exploring the potential capability of Nqo15 to bind iron. An analogous case has also been reported for the YdhG protein of *Bacillus subtilis* [43,59], which, despite its low sequence similarity with the members of the FXN family, has a structure that superimposes with the peculiar α-β motif; the RMSD between the NMR solution structure of FXN (PDB ID: 1LY7) and this protein (PDB ID: 2OC6) is 3.1 Å, while it is 3.4 Å between FXN and Nqo15. In YdhG, several Asp and Glu residues are distributed along the first α-helix and the first β-strand, potentially involved in iron-binding; additionally, the authors demonstrate that YdhG interacts with IscU-type Fe-S cluster assembly proteins and stimulates the formation of Fe-S clusters. The case of Nqo15 could differ from that of YdhG, since, although structurally similar to members of the FXN family, Nqo15 is an integral part of a protein complex. When comparing the iron-binding properties of Nqo15 and human FXN, we have already mentioned the different charges of the two proteins, acidic for FXN and basic for Nqo15. Fluorescence spectroscopy, NMR and the iron (III)-binding assay clearly show that Nqo15 is not able to bind iron or keep it in a bioavailable form at a neutral pH, at least in vitro (Figure 5). Thus, our results show that if Nqo15 plays an iron-trafficking and/or iron-storage role, it does so only when bound to complex I, since such a property is lost if isolated. For this reason, it must be questioned whether the affinity of Nqo15 for iron might be modulated when the protein is bound to complex I. In this regard, it is worth noting that the negatively charged residues of Nqo15 placed in the N-terminal α-helix (i.e., Asp and Glu residues, Figure 5A in red), also highly conserved among the members of the FXN family, are not directly involved in interactions with the adjacent subunits of the hydrophilic domain in complex I. This allows us to speculate that even if stably bound to complex I, the interaction of Nqo15 with iron could still start through the negatively charged α-helix, which remains exposed to the solution. Furthermore, it should be noted that the His residues of Nqo15 that line the putative iron-binding channel in *T. thermophilus* are completely absent in the β-sheet region of FXN (Figure 5A), ascribing this peculiar feature only to Nqo15.

In addition to iron trafficking, FXN has been reported to participate in the biogenesis of Fe-S clusters as an allosteric activator for NFS1 [19,20,21,22,23,24]; this function, involving Fe-S clusters, could have been another common feature of the two proteins. Here, however, we confirm that the capability of human FXN to activate desulfurase is not shared by Nqo15 (Figure 6); indeed, Nqo15 lacks the tryptophan residue critical in FXN for the formation of the protein complex involved in the biogenesis of Fe-S groups, i.e., W155 [60,61,62].

## 4. Materials and Methods

### 4.1. Protein Expression and Purification

The coding sequence for *T. thermophilus* Nqo15 was cloned into a pET-9b plasmid vector (Novagen) suitable for T7-driven expression in *E. coli*. Molecular cloning was performed with the In-Fusion HD Cloning Kit (Takara Bio Inc.) according to the manufacturer’s guidelines. The *Nqo15* insert and the linearized pET-9b vector were previously amplified by PCR, starting, respectively, from a pUC57 vector containing the *Nqo15* sequence (GenScript Biotech, Piscataway, NJ, USA) and a circularized pET-9b vector, using the following designed primers:

Fw_Nqo15_: 5′-GGAGATATACATATGAGCGCGTCTTCCGAGCGCGAACTC-3′;
Rev_Nqo15_: 5′-GCAGCCGGATCCTCAGGCGAAGGCCAAAGCCTCCCGC-3′;
Fw_pET-9b_: 5′-TGAGGATCCGGCTGCTAACAAAG-3′;
Rev_pET-9b_: 5′-CATATGTATATCTCCTTCTTAAAG-3′.

The final construct (*pet-9b/Nqo15*) was analyzed by DNA sequencing (BMR Genomics, University of Padova) and used to transform *E. coli* BL21 (DE3) cells, selecting positive clones by antibiotic resistance. Bacteria cultures were grown at 37 °C and 180 rpm in Luria–Bertani Broth or M9 minimal media supplemented with ^15^N NH_4_Cl for the isolation of the ^15^N-labeled protein used in the NMR experiments. Protein expression was induced at OD_600nm_ = 0.5 by the addition of 1 mM isopropyl-β-thiogalactopyranoside (IPTG) and incubating the bacteria cultures at 30 °C overnight under constant stirring (170 rpm). After induction, bacteria were centrifuged at 5000× *g*, 4 °C for 15 min and the pellet was stored at −20 °C until further use. The cell pellet was resuspended in lysis buffer, 25 mM Tris–HCl (pH 9.5) supplemented with protease inhibitors (1.0 μg/mL pepstatin A, 1.0 μg/mL leupeptin, 1.0 μg/mL antipain, 100 μM PMSF), and lysed by multiple cycles of sonication. Soluble and insoluble fractions were separated by centrifugation at 13,300× *g*, 4 °C for 15 min. The soluble fraction was then incubated with 10 mM EDTA under gentle agitation, at 4 °C, for at least 1 h. The protein was purified by combining anionic exchange chromatography (AEC) and size-exclusion chromatography (SEC). Briefly, the first chromatographic step was performed by incubating the supernatant with 10 mL of DEAE Sepharose™ resin (Cytiva, Amersham, UK) for 1 h, under gentle agitation, at 4 °C. After the removal of the flow through, an elution buffer (25 mM Tris–HCl, 50 mM KCl, pH 9.5) was added to the resin and the fractions containing the protein, as assessed by SDS-PAGE, were collected, pooled together and concentrated by centrifugal filters (Amicon Ultra Centrifugal Filter, 3000 NMWL, from Merck, Burlington, MA, USA) to a final volume suitable for the next chromatographic step. The protein was then purified by SEC using a Superdex 200 GL 10/300 column (from Cytiva, Amersham, UK), equilibrated in a buffer containing 25 mM HEPES, 50 mM KCl, pH 6.5. The eluted fractions containing Nqo15 were finally pooled together, frozen and stored at −20 °C until further use. The molar concentration of the protein samples was determined spectroscopically using ε_280nm_ = 18,450 M^−1^cm^−1^ as evaluated computationally by the ExPASy webserver, ProtParam tool. Protein purity and integrity were assessed by 4–20% SDS-PAGE (GenScript^®^ polyacrylamide gel, GenScript Biotech, Piscataway, NJ, USA) and Coomassie blue staining, prior to any spectroscopic experiment reported in this work.

The heterologous expression and purification of human FXN (residues 90–210), ISCU and complex NFS1/ACP-ISD11 were performed as previously described [63].

### 4.2. Static Light Scattering (SLS) Measurements

Molecular mass determination was performed by multiple-angle laser light scattering (MALLS) using a miniDawn instrument (Wyatt Technology, Santa Barbara, CA, USA) for static measurements, in conjunction with a size-exclusion Superose-12 column (GE Healthcare). The protein concentration was 260 μM, and the elution buffer consisted of 20 mM Tris–HCl and either 50 mM or 200 mM NaCl, pH 7.0. The experiment was carried out at room temperature (25 °C) with a flow rate of 0.4 mL/min. Data analysis was conducted using the Astra 6.0 software (Wyatt Technology).

### 4.3. Circular Dichroism (CD) Spectroscopy

CD measurements were performed with a Jasco J-1500 spectropolarimeter equipped with a Jasco PTC-510 Peltier cell holder connected to a Jasco PTC-423S Peltier controller. Far-UV CD spectra were collected using a cylindrical cell (121-0.20-40, Hellma, Milan, Italy) with a 0.2 mm optical path length using 50 μL of protein solution at a concentration of 30 μM in the following buffer: 10 mM phosphate buffer, pH 6.5. Experiments were performed from 25 to 80 °C (sample holder temperature), keeping the cuvette compartment under a constant nitrogen flow. Data were acquired in the 260–180 nm interval every 0.5 nm using the step scan method with an adaptive integration time (1 to 8 s); two to four scans were averaged.

### 4.4. Nuclear Magnetic Resonance (NMR) Spectroscopy

NMR experiments were performed on a Bruker AVANCE NEO 600 MHz spectrometer, equipped with a 5 mm cryogenic probe, the Prodigy TCI. ^1^H-^15^N SOFAST HMQC spectra [47] were acquired at 25 °C on 50 μM ^15^N-labeled Nqo15 samples in a buffer of 25 mM HEPES, 50 mM KCl, pH 6.5, with the addition of 10% D_2_O up to a final volume of 550 μL. In the experiments with iron, 200 µM Fe^2+^ solution was used (1:4 Nqo15:Fe^2+^ ratio). For Fe^2+^ addition, a ferrous solution (50 mM) was previously prepared in an anaerobic glove box by dissolving Mohr’s salt (NH_4_)_2_Fe(SO_4_)_2_·6H_2_O in a N_2_-purged buffer containing 25 mM HEPES, 50 mM KCl, pH 6.5. Before each NMR experiment, the pH of all samples was measured and, if necessary, adjusted with HCl to 6.5. Samples were then transferred to standard NMR tubes and sealed with appropriate rubber septa.

### 4.5. Fluorescence Spectroscopy

Fluorescence experiments were performed on a FLS 1000 UV/Vis/NIR photoluminescence spectrometer (Edinburgh Instruments Ltd., Livingston, UK) with a 450 W Xenon Arc lamp for excitation at 280 nm and a PMT-980 detector. The excitation wavelength was set at 280 nm and emission was recorded in the range between 285 and 500 nm. Measurements were performed at 15 °C, under constant stirring, and the sample compartment was kept under nitrogen flow to avoid condensation and to ensure the anaerobic conditions. Experiments were performed using quartz cuvettes (117104F-10-40, Hellma, Milan, Italy) with a 10 × 4 mm optical path length and a gas-tight screw cap with a silicon septum for the addition of the ferrous/ferric iron solutions via a gas-tight microsyringe (Hamilton Company, Reno, NV, USA). Samples were directly prepared in the cuvette, diluting the protein to a final concentration of 2.2 μM in a buffer of 25 mM HEPES, 50 mM KCl, pH 6.5. Titration with Fe^2+^ was performed using a 165 μM ferrous solution obtained from a dilution of a 50 mM solution previously prepared dissolving Mohr’s salt in a N_2_ purged buffer of 25 mM HEPES, 50 mM KCl, pH 6.5. Sample preparation and measurement recording were performed in strictly anaerobic conditions to prevent Fe^2+^ oxidation.

### 4.6. Iron (III)-Binding Capability Assay

Protein samples were extensively buffer-exchanged to 50 mM HEPES and 50 mM NaCl at pH 7.0 using Amicon 4 devices (Merck, Burlington, MA, USA). Microcentrifuge tubes containing human FXN (residues 90 to 210) or Nqo15 were preincubated at 25 °C and then FeCl_3_ was added from a stock solution to achieve final concentrations of 50 μM for the protein and 250 μM for iron in a total volume of 150 μL. Immediately after the addition of FeCl_3_, one sample was taken to analyze iron without centrifugation (time zero, total iron) and the other sample was centrifuged for 5 min at 20,000× *g* at 25 °C, followed by the quantification of soluble iron. The iron concentration was determined by the 1,10-phenanthroline method [64]. Briefly, a 100 μL sample of the supernatant was combined with 100 μL of 10% sodium citrate, 100 μL of 10% ascorbic acid, 100 μL of 0.25% 1,10-phenanthroline and 600 μL of mQ water. After incubation for 30 min at room temperature, the samples were centrifuged at 20,000× *g* for 10 min and the absorbance was measured at 512 nm. The absorbance values were then converted into iron concentrations using a standard calibration curve.

### 4.7. Cysteine Desulfurase Activity Measurements

Enzyme activity was monitored by sulfide production using a previously described assay [52], with minor modifications. Protein samples in 50 mM Tris–HCl, 200 mM NaCl, pH 8.0 were supplemented with 10 μM pyridoxal phosphate (PLP), 2 mM dithiothreitol (DTT) and 1 μM Fe (II). Reactions contained 1 μM NFS1/ACP-ISD11, 3 μM ISCU and 3 μM human FXN (residues 90–210) or Nqo15. To initiate the reaction, 1.0 mM cysteine was added (the total reaction volume was 400 μL) and samples were incubated at room temperature for 30 min. The generated sulfide was then quantified by the methylene blue method [53]. To this end, the reaction was stopped by the addition of 50 μL of 20 mM *N*,*N*-dimethyl p-phenylenediamine (DMPD) in 7.2 N HCl and 50 μL of 30 mM FeCl_3_ prepared in 1.2 N HCl. After 20 min incubation at room temperature in the dark, the samples were centrifuged at 12,000× *g* for 5 min and the absorbance was measured at 670 nm.

### 4.8. In Silico Methods

The crystal structure of Nqo15 (resolution: 3.30 Å) was extracted from PDB.ID 2FUG corresponding to the respiratory complex I structure coming from *T. thermophilus* [41]. Nqo15 corresponds to the 7-H-Q-Z chains of the crystal structure. In order to search for evolutionary relationships between FXN and Nqo15, sequence- and structural-based methods were run. BLAST, PSIBLAST and HMMER [65] were used to explore sequence relationships using canonical human FXN and Nqo15 sequences. For structural-based methods, the FoldSeek [66] and Dali [67] servers were used. Different database searches were performed using SCOP (a structural-based classification of proteins with known crystallographic structure [68]) and Interpro (a sequence- and structure-based classification of proteins [69]). Finally, structural-based alignments were derived using Bio3D for R [70]. The structural flexibility was explored using coarse-grained molecular dynamics simulations (MD) performed on the CABS-flex 2.0 webserver [71] using standard parameters on the structure of chain H. The CD data were analyzed using the tools available in the BeStSel webserver [48]: we predicted the secondary structure content based on both the experimental CD spectrum and the crystal structure. The prediction of the CD spectrum from the MD simulations was performed using the PDBMD2CD webserver [49].

## 5. Conclusions

The ability of Nqo15 to improve the respiratory phenotype of FRDA patient cells, while lacking desulfurase activation and iron-binding in vitro, suggests that the similarity of the two proteins is centered on a common ability to interact with complex I in a way that is yet to be defined. We consider that the details of this interaction are worthy of further exploration, and this is definitely possible since we have demonstrated that Nqo15 can be recombinantly expressed in a stable, soluble and monomeric form, retaining its globular structure also when isolated from complex I. Further analysis based on structural divergence and dynamical patterns could shed light on additional roles of FXN. The analysis of disease-associated variants could also contribute to this goal.

## Figures and Tables

**Table 1:** Structural superfamily members as described by SCOP database.

Family	Organism	Representative PDB ID
Frataxin-like	*Saccharomyces cerevisiae* S288C	5TRE_C
Frataxin-like	*Saccharomyces cerevisiae* S288C	2FQL_A
Frataxin-like	*Saccharomyces cerevisiae* S288C	4EC2_A
Frataxin-like	*Saccharomyces cerevisiae* S288C	3OER_A
Frataxin-like	*Homo sapiens*	3S5F_A
Frataxin-like	*Homo sapiens*	3T3T_C
Frataxin-like	*Homo sapiens*	1EKG_A
Frataxin-like	*Homo sapiens*	6NZU_I
Frataxin-like	*Homo sapiens*	3S4M_A
Frataxin-like	*Psychromonas ingrahamii* 37	4HS5_A
Frataxin-like	*Psychromonas ingrahamii* 37	4LK8_A
Frataxin-like	*Psychromonas ingrahamii* 37	4LP1_A
Frataxin-like	*Chaetomium thermophilum var. thermophilum* DSM 1495	6FCO_C
Frataxin-like	*Escherichia coli* K-12	1EW4_A
Frataxin-like	*Escherichia coli* K-12	2EFF_A
Frataxin-like	*Escherichia coli* K-12	2P1X_A
Frataxin-like	*Escherichia coli* K-12	1SOY_A
Frataxin-like	*Burkholderia cenocepacia* J2315	4JPD_A
Nqo15-like	*Thermus thermophilus* HB8	2ZJL_7
Nqo15-like	*Thermus thermophilus* HB8	6I0D_I
Nqo15-like	*Thermus thermophilus* HB8	6Q8X_7
Nqo15-like	*Thermus thermophilus* HB8	6I1P_7
Nqo15-like	*Thermus thermophilus* HB8	6I1P_I
Nqo15-like	*Thermus thermophilus* HB8	6Y11_I
Nqo15-like	*Thermus thermophilus* HB8	3IAS_Q
Nqo15-like	*Thermus thermophilus* HB8	2FUG_Q
Nqo15-like	*Thermus thermophilus* HB8	3M9S_7
Nqo15-like	*Thermus thermophilus* HB8	3IAM_7
Nqo15-like	*Thermus thermophilus* HB8	4HEA_7
Nqo15-like	*Thermus thermophilus* HB8	6Y11_7
YdhG-like	*Lactobacillus paracasei ATCC 334*	2I8D_A
YdhG-like	*Bacillus subtilis subsp. subtilis str. 168*	2OC6_A
YdhG-like	*Bacillus halodurans C-125*	2KL4_A

## Data Availability

Raw data can be provided upon request to the corresponding authors.
